# Tachycardia-Mediated Cardiomyopathy from Concealed Left-Sided AVRT with Complete Recovery Post-Ablation

**DOI:** 10.1093/omcr/omag080

**Published:** 2026-05-24

**Authors:** Yi Lung Gan, Vinay Kumar, Hameed Ullah, Anwar Hussain, Manish Kalla, Purushottam Desai

**Affiliations:** Department of Cardiology, Queen Elizabeth Hospital Birmingham, Mindelsohn Way, Birmingham, B15 2GW, United Kingdom; Department of Medicine, Walsall Healthcare NHS Trust, Moat Road, Walsall, West Midlands, WS2 9PS, United Kingdom; Department of Cardiology, Queen Elizabeth Hospital Birmingham, Mindelsohn Way, Birmingham, B15 2GW, United Kingdom; Department of Cardiology, Queen Elizabeth Hospital Birmingham, Mindelsohn Way, Birmingham, B15 2GW, United Kingdom; Department of Cardiology, Queen Elizabeth Hospital Birmingham, Mindelsohn Way, Birmingham, B15 2GW, United Kingdom; Department of Cardiology, Queen Elizabeth Hospital Birmingham, Mindelsohn Way, Birmingham, B15 2GW, United Kingdom

**Keywords:** tachycardia mediated cardiomyopathy, atrioventricular reentrant tachycardia, heart failure, congestive hepatopathy, concealed left sided accessory pathway

## Abstract

Tachycardia mediated cardiomyopathy (TMC) is a reversible form of cardiomyopathy that results from persistent or incessant tachyarrhythmias. TMC may progress rapidly and present with symptoms of decompensated heart failure, cardiogenic shock, or multisystem organ dysfunction, sometimes in individuals with no prior cardiac history. Prompt recognition of the arrhythmia and early elimination of the underlying tachycardia are crucial, as restoration of sinus rhythm typically leads to complete or near-complete recovery of ventricular function [1]. Incessant supra-ventricular tachycardia (SVT) can be challenging to diagnose when patients present with advanced heart failure and multiorgan involvement and misinterpretation may delay definitive therapy. This case highlights an extreme presentation of incessant orthodromic AVRT causing biventricular failure, severe hepatic congestion and metabolic acidosis, with complete recovery following catheter ablation of a concealed left-sided accessory pathway.

## Case presentation

A 49-year-old gentleman with no known medical illness presented with 2 weeks history of palpitation, shortness of breath and leg swelling. He denied any pre-existing chest pain or previous palpitations. On initial examination, his heart rate was 135 beats per minute, blood pressure 130/90 mmHg and oxygen saturation was 90% requiring high flow nasal oxygen. He was deeply jaundiced with elevated jugular venous pressure and peripheral oedema to suggest fluid overload. Cardiovascular examination revealed normal heart sounds and respiratory examination showed bilateral crepitations.

He suffered a short period of respiratory arrest and initial blood gas showed severe metabolic acidosis hence was transferred to intensive care unit for respiratory support and later continuous renal replacement therapy via hemodiafiltration due to severe metabolic acidosis and fluid overload.

His blood investigations showed significant liver impairment with alanine transaminase (ALT) of 1226 U/L and total bilirubin of 233 umol/L suggestive of hepatic congestion. Other blood investigations are outlined as below in Fig. 1. Chest radiograph showed florid pulmonary oedema with bilateral pleural effusions. (Fig. 2) Electrocardiogram (ECG) showed tachycardia with right bundle branch block with a long RP interval (Fig. 3). A computed tomography (CT) scan was done for thorax, abdomen and pelvis which showed evidence of ascites and pleural effusion with dilated right atrium and ventricle with evidence of contrast reflux into the hepatic veins suggestive of volume overload. A transthoracic echocardiogram with limited image quality due to patient’s body habitus showed a left ventricle with severely impaired systolic function and an impaired right ventricular function (Fig. 4).

**Figure 1:**
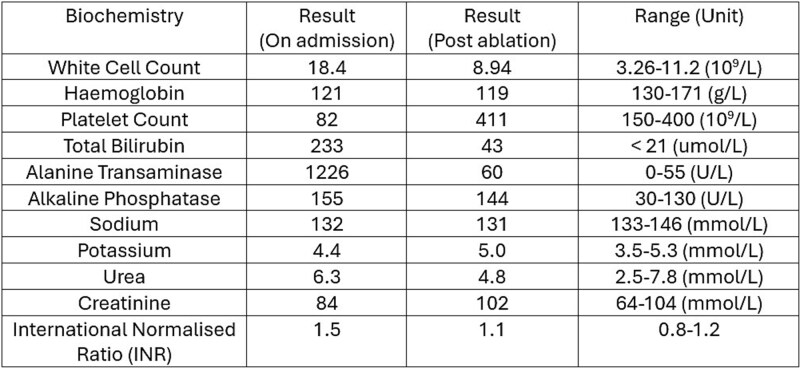
Blood investigations depicting severe liver impairment which improved following treatment of tachycardia mediated cardiomyopathy.

**Figure 2:**
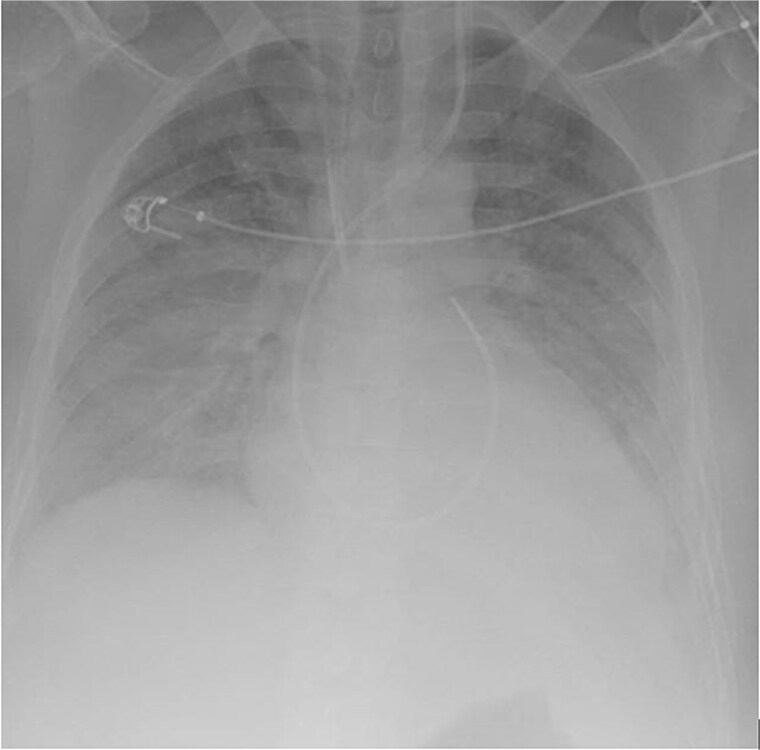
Chest radiograph showing pulmonary oedema with pulmonary artery catheter via the left internal jugular vein and the dialysis catheter via the right internal jugular vein.

**Figure 3:**
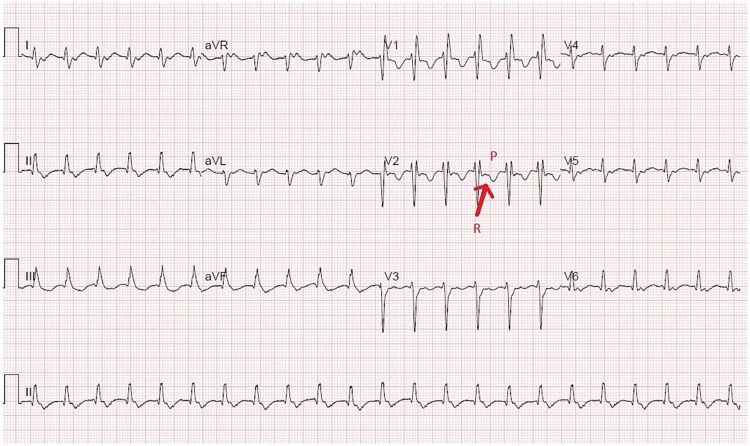
Electrocardiogram (ECG) showing long RP tachycardia with right bundle branch block (RBBB) morphology.

**Figure 4:**
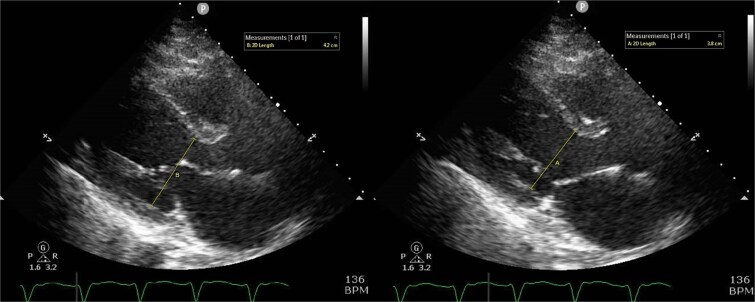
Parasternal long axis view of transthoracic echocardiogram depicting fractional shortening of 9.7% suggestive of severe left ventricular systolic impairment. (Before ablation).

He was treated as likely tachycardia mediated cardiomyopathy causing biventricular failure. He had direct current cardioversion, was given digoxin and started on amiodarone infusion in spite of which unfortunately the tachycardia remained incessant. He did have periods of restoration of sinus rhythm with administration of adenosine however quickly went back into tachycardia. He required milrinone infusion whilst on intensive care to support his blood pressure.

Given his acute presentation with no other clear aetiology, he was discussed in the cardiology multidisciplinary team (MDT) meeting and a decision was made for an urgent inpatient electrophysiology study (EPS). A 3 wires EPS was performed via the right femoral vein. A decapolar catheter was put in the coronary sinus (CS) and a quadripolar catheter was used to map the His signal. He was in incessant tachycardia during the study which the coronary sinus catheter showed eccentric activation with earliest activation at the distal pole. Ventricular overdrive pacing confirmed a V-A-V response with short post pacing interval. This effectively ruled out other differentials of long RP tachycardia as atrial tachycardia should have a V-A-A-V response while atrio-ventricular nodal re-entry tachycardia (AVNRT) and permanent junctional reciprocating tachycardia (PJRT) should not demonstrate eccentric CS activation. A trans-septal puncture was performed with heparin cover through which then an ablation catheter was passed into the left side and the earliest atrial electograms were mapped in tachycardia. This confirmed an orthodromic atrioventricular reciprocating tachycardia (AVRT) with a concealed left sided pathway around 5 o’clock of the mitral annulus. The pathway was ablated with radiofrequency energy at 40 W as shown in Fig. 5. Endpoint was determined by immediate termination of tachycardia, the absence of ventriculo-atrial (VA) conduction during retrograde pacing and non-inducible tachycardia after the ablation.

**Figure 5:**
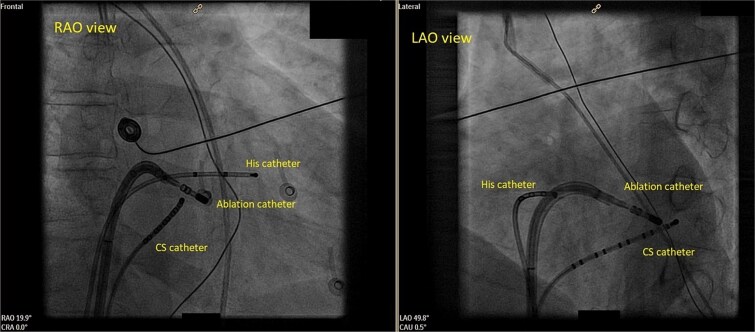
Fluoroscopy during EP study showing biplane imaging in the position of right anterior oblique (RAO) and left anterior oblique (LAO) with three catheters placement from the right femoral vein. The ablation catheter is trans-septal through the inter-atrial septum and placed at the posterior mitral annulus around 5 o’clock position (position confirmed with the earliest atrial activation on the ablation catheter’s electograms). Ablation with radiofrequency here immediately terminated the tachycardia.

Following ablation, the patient had a repeat transthoracic echocardiography in 2 days which showed significant improvement in his biventricular systolic function with a left ventricular ejection fraction (EF) of 52% and a normal right ventricular systolic function. (Fig. 6) His blood investigation also showed significant improvement in his liver function test. (Fig. 1) His pre-discharge ECG showed sinus rhythm with right bundle branch block morphology. (Fig. 7) As he remained well with almost normalisation of his biventricular function and no cardiac or family history to suggest a primary cardiomyopathy, we felt that an MRI is not needed. He was discharged well with subsequent follow-up in the outpatient cardiology clinic.

**Figure 6:**
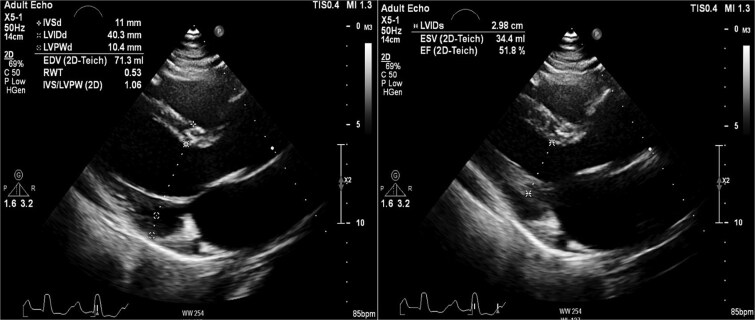
Parasternal long axis view of transthoracic echocardiogram showing improvement in left ventricular systolic function with a 2D ejection fraction of 52% and fractional shortening of 26% post ablation (Improved from 9.7%).

**Figure 7:**
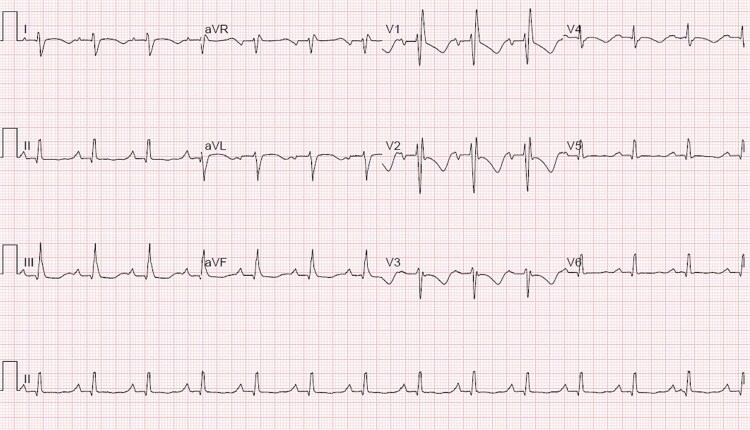
Post ablation and pre-discharge ECG showing restoration of sinus rhythm with RBBB morphology.

## Discussion

This case demonstrates an uncommon but important presentation of incessant orthodromic atrioventricular reciprocating tachycardia (AVRT) leading to severe tachycardia mediated cardiomyopathy (TMC) with biventricular failure, multi-organ dysfunction, and haemodynamic collapse requiring intensive care support. The immediate improvement following catheter ablation highlights the reversibility of TMC when the arrhythmia substrate is promptly eliminated.

TMC is a reversible cause of heart failure resulting from persistent tachyarrhythmia, most classically atrial fibrillation and rarely incessant supraventricular tachycardias such as AVRT [1–3]. It typically develops over weeks to months. The pathophysiology is due to progressive left ventricular (LV) dilatation and systolic impairment with reduction of LV dP/dt_max_ and myocardial blood flow. As a result, this increases the LV wall stress and end-diastolic pressure and volume which lead to the cascade of heart failure clinical manifestation [4]. Conduction abnormalities may also cause heart failure symptoms such as AV dyssynchrony in those with significant first-degree AV block or ventriculo-ventricular (VV) dyssynchrony due to bundle branch block causing inter and intraventricular conduction delays. More often it is the left bundle branch block (LBBB) rather than right bundle branch block (RBBB) which is associated with heart failure and this is due to LBBB causing LV dyssynchrony which directly affects LV systolic function [5]. In this case, the patient presented with only a short history of symptoms, yet already had severe biventricular systolic dysfunction, pulmonary oedema, hepatic congestion, and severe metabolic acidosis requiring renal replacement therapy.

The initial ECG showed a long RP tachycardia, which includes differentials such as atypical AVNRT, permanent junctional reciprocating tachycardia (PJRT), atrial tachycardia, or orthodromic AVRT with a concealed accessory pathway. The patient’s response to adenosine with transient termination with rapid re-initiation is characteristic of re-entrant tachycardias via the AV node with an easily inducible substrate [6].

The patient developed multiorgan dysfunction including acute liver injury (ALT > 1200 U/L), hyperbilirubinemia, metabolic acidosis, and respiratory failure. Severe heart failure with congestive hepatopathy is well described, with biochemical patterns ranging from cholestasis to dramatic transaminase elevations in acute cardiogenic shock [7, 8]. Similarly, acute kidney injury and metabolic acidosis occur frequently in low-output states from reduced renal perfusion and venous congestion [9]. Prompt recognition of the arrhythmic origin of these abnormalities is crucial because treatment of the tachycardia reverses the underlying hemodynamic insult, as demonstrated here.

Left-sided concealed pathways as demonstrated in our patient are well recognized causes of orthodromic AVRT [10]. Radiofrequency catheter ablation is considered first-line therapy for symptomatic or incessant AVRT and has an acute success rates exceeding 95% for left-sided pathways [11].

In this case, despite medical therapy including digoxin, amiodarone, adenosine, and DC cardioversion, the tachycardia remained incessant. In such scenarios, early referral for EPS is essential because ongoing tachycardia drives further deterioration. Ablation resulted in immediate non-inducibility and termination of VA conduction which are established endpoints indicating procedural success.

The most striking feature of this case is the rapid recovery of both left and right ventricular systolic function within 48 hours of ablation. TMC primarily affects the LV, but RV involvement can occur in chronic or severe cases [12]. The patient’s dramatic improvement strongly supports TMC rather than a primary cardiomyopathic process. Studies showed that normalization of LV ejection fraction may occur within days to weeks after elimination of the tachyarrhythmia, although full reverse remodeling may take several months [13]. Early improvement following ablation predicts excellent long-term outcomes.

## Conclusion

This case demonstrates that incessant tachycardia can lead to profound biventricular failure and multiorgan dysfunction even in previously healthy individuals. Tachycardia mediated cardiomyopathy should always be a differential diagnosis in patients with unexplained cardiac failure and incessant tachyarrhythmia. When medical therapy fails to terminate the tachycardia or when organ dysfunction progresses, early electrophysiological evaluation and catheter ablation are essential. Successful elimination of the tachycardia can result in rapid and complete reversal of myocardial dysfunction, highlighting the importance of early recognition and definitive management of tachycardia mediated cardiomyopathy.

### Learning Points

1) Incessant tachycardia can lead to rapidly progressive tachycardia-mediated cardiomyopathy which may present with biventricular failure and multiorgan dysfunction such as hepatic congestion, renal impairment and metabolic acidosis.
2) Failure of medical therapy to control the tachycardia should prompt early electrophysiology intervention, as catheter ablation is highly effective and often curative, particularly for AVRT secondary to accessory pathways.
3) Tachycardia-mediated cardiomyopathy is highly reversible, and prompt restoration of sinus rhythm can lead to rapid normalization of ventricular function and improvement in clinical condition.

## References

[1] Medi C, Kalman JM, Haqqani H. et al. Tachycardia-mediated cardiomyopathy secondary to focal atrial tachycardia: long-term outcome after catheter ablation. Heart Rhythm 2009;6:188–93. 10.1016/j.jacc.2009.02.014 19422986

[2] Shinbane JS, Wood MA, Jensen DN. et al. Tachycardia-induced cardiomyopathy: a review of animal models and clinical studies. J Am Coll Cardiol 1997;29:709–15. 10.1016/S0735-1097(96)00592-X 9091514

[3] Gopinathannair R, Etheridge SP, Marchlinski FE. et al. Arrhythmia-induced cardiomyopathies: mechanisms, recognition, and management. J Am Coll Cardiol 2015;66:1714–28. 10.1016/j.jacc.2015.08.038 26449143 PMC4733572

[4] Huizar JF, Ellenbogen KA, Tan AY. et al. Arrhythmia-induced cardiomyopathy: JACC state-of-the-art review. J Am Coll Cardiol 2019;73:2328–44. 10.1016/j.jacc.2019.02.045 31072578 PMC6538508

[5] Huizar JF, Kaszala K, Tan A. et al. Abnormal conduction-induced cardiomyopathy: JACC review topic of the week. J Am Coll Cardiol 2023;81:1192–200. 10.1016/j.jacc.2023.01.040 36948737 PMC10715964

[6] Knight BP, Ebinger M, Oral H. et al. Diagnostic value of the response to adenosine in patients with narrow QRS supraventricular tachycardia. J Am Coll Cardiol 2000;36:2035–40. 10.1016/s0735-1097(00)00770-1 10933374

[7] Alvarez AM, Mukherjee D. Liver abnormalities in cardiac diseases and heart failure. Int J Angiol 2011;20:135–42. 10.1055/s-0031-1284434 22942628 PMC3331650

[8] Myers RP, Cerini R, Sayegh R. et al. Cardiac hepatopathy: clinical, hemodynamic, and histologic characteristics and correlations. Hepatology. 2003;37:393–400. 10.1053/jhep.2003.50062 12540790

[9] Mullens W, Abrahams Z, Francis GS. et al. Importance of venous congestion for worsening of renal function in heart failure. J Am Coll Cardiol 2009;53:589–96. 10.1016/j.jacc.2008.05.068 19215833 PMC2856960

[10] Katritsis DG, Merchant FM, Mela T. et al. Concealed accessory pathways: mechanisms and clinical implications. Europace. 2012;14:1110–8.

[11] Page RL, Joglar JA, Caldwell MA. et al. 2015 ACC/AHA/HRS SVT guideline. Circulation. 2016;133:e506–74. 10.1161/CIR.0000000000000311 26399663

[12] Fenelon G, Wijns W, Andries E. et al. Tachycardiomyopathy: mechanisms and clinical implications. Pacing Clin Electrophysiol 1996;19:95–106. 10.1111/j.1540-8159.1996.tb04796.x 8848384

[13] Hasdemir C, Yuksel A, Camli D. et al. Tachycardia-induced cardiomyopathy in patients with idiopathic ventricular arrhythmias: the incidence, clinical and electrophysiologic characteristics, and the predictors. Int J Cardiol 2014;176:1207–12. 21235667 10.1111/j.1540-8167.2010.01986.x

